# HSulf-2, an extracellular endoglucosamine-6-sulfatase, selectively mobilizes heparin-bound growth factors and chemokines: effects on VEGF, FGF-1, and SDF-1

**DOI:** 10.1186/1471-2091-7-2

**Published:** 2006-01-17

**Authors:** Kenji Uchimura, Megumi Morimoto-Tomita, Annette Bistrup, Jessica Li, Malcolm Lyon, John Gallagher, Zena Werb, Steven D Rosen

**Affiliations:** 1 Department of Anatomy and the UCSF Comprehensive Cancer Center, University of California, San Francisco, CA 94143-0452, USA; 2 Thios Pharmaceuticals, 5980 Horton Street, Emeryville, CA 94608, USA; 3 Department of Medical Oncology, University of Manchester, Paterson Institute for Cancer Research, Manchester, UK

## Abstract

**Background:**

Heparin/heparan sulfate (HS) proteoglycans are found in the extracellular matrix (ECM) and on the cell surface. A considerable body of evidence has established that heparin and heparan sulfate proteoglycans (HSPGs) interact with numerous protein ligands including fibroblast growth factors, vascular endothelial growth factor (VEGF), cytokines, and chemokines. These interactions are highly dependent upon the pattern of sulfation modifications within the glycosaminoglycan chains. We previously cloned a cDNA encoding a novel human endosulfatase, HSulf-2, which removes 6-O-sulfate groups on glucosamine from subregions of intact heparin. Here, we have employed both recombinant HSulf-2 and the native enzyme from conditioned medium of the MCF-7-breast carcinoma cell line. To determine whether HSulf-2 modulates the interactions between heparin-binding factors and heparin, we developed an ELISA, in which soluble factors were allowed to bind to immobilized heparin.

**Results:**

Our results show that the binding of VEGF, FGF-1, and certain chemokines (SDF-1 and SLC) to immobilized heparin was abolished or greatly diminished by pre-treating the heparin with HSulf-2. Furthermore, HSulf-2 released these soluble proteins from their association with heparin. Native Sulf-2 from MCF-7 cells reproduced all of these activities.

**Conclusion:**

Our results validate Sulf-2 as a new tool for deciphering the sulfation requirements in the interaction of protein ligands with heparin/HSPGs and expand the range of potential biological activities of this enzyme.

## Background

Heparan sulfate proteoglycans (HSPGs) abundantly exist in the ECM and on the cell surface of most cells [1,2]. They are composed of a restricted set of core proteins to which one or more glycosaminoglycan chains (GAG) are covalently attached. It is established that HSPGs are involved in a wide range of biological functions through their ability to bind to and modify the activities of a diverse repertoire of ligands including growth factors, morphogens, cytokines, chemokines, proteases, lipases, matrix proteins, and cell adhesion molecules [3-6]. Heparan sulfate (HS) chains and heparins (chemical analogues of these chains) consist of repeating disaccharide units of glucuronic/iduronic acid and glucosamine that are subject to a complex set of modifications involving deacetylation, epimerization, and sulfation. Four different sites of sulfation are found in heparin/HS: the N-, 3-O, and 6-O positions of glucosamine and the 2-O position of the iduronic acid residue. Heparin is highly-sulfated throughout the polymer chain whereas in HS these modifications are concentrated mainly in the S-domains, which consist of contiguous clusters of N-sulfated disaccharide units variably sulfated at the other positions [7-9]. Interspersed with the S domains are regions with low (transition zones) or zero sulfation; however, the transition zones contain a considerable fraction of the 6-O-sulfates in HS [10]. The ligand binding activities of HSPG/heparin depend on patterns of sulfation along the chains [2].

HS chains are dynamically regulated in development and during tumor progression [11,12]. Since these changes are central to the ligand binding properties of HSPGs, there is considerable interest in mechanisms that generate diversity of the chains. One such mechanism is through regulated expression of enzymes involved in the biosynthesis of heparan sulfate, for example, the sulfotransferases that modify HS chains [13]. Another potential mechanism is through the action of extracellular endosulfatases that remove specific sulfation modifications from intact GAG chains. The first enzyme identified in this category was QSulf-1, which was discovered in quail embryo [14]. Stimulated by this work, we cloned cDNAs encoding Sulf-1 and a new member of the family, Sulf-2, in mouse and human [15]. We showed that both Sulf-1 and Sulf-2 are secreted into conditioned medium when they are expressed in Chinese hamster ovary (CHO) cells. Both possess endoglucosamine-6-sulfatase activity against intact heparin with an optimum at neutral pH. The enzymes liberate sulfate groups from the C-6 position of glucosamine residues on trisulfated -IdoA(2-OSO_3_)-GlcNSO_3_(6-OSO_3_)- disaccharide units of heparin. A similar activity for QSulf-1 has now been confirmed on HS chains [16,17]. This trisulfated disaccharide structure occurs within the S-domains and is known to be a key element in many of the protein ligand interactions of heparin and HS (see below).

The transcripts corresponding to QSulf-1 and its rat orthologue (RFP-Sulf-1) demonstrate complex spatiotemporal regulation during embryonic development [14,18] and QSulf-1 plays an essential role in Wnt-dependent differentiation of somites into muscle in quail [14]. In addition, in vitro assays have demonstrated that QSulf-1 promotes both Wnt [14] and bone morphogenetic protein [17] signaling via its sulfatase activity. Less is known about the distribution and function of the Sulfs in adult tissues. However, some interesting correlations have been revealed in tumors. Lai and colleagues have reported downregulation of Sulf-1 transcripts in human ovarian cancer and a subset of hepatocellular carcinomas [19-21]. These workers and others have stressed the potential role of the enzyme in down-modulating certain signaling pathways involved in cell proliferation, since over-expression of Sulf-1 reduces signaling by FGF-2, HB-EGF, or HGF [19-22]. In striking contrast to the results in ovarian cancer, increased levels of *SULF1 *or *SULF2 *transcripts are observed in other human cancers including breast and pancreatic carcinomas [23-25]. Upregulation of Sulf-2 at both the transcript and protein levels has been established in two mouse models of mammary carcinoma [25]. Furthermore, cultured human breast carcinoma cells release enzymatically active Sulf-2 into conditioned medium. The upregulation of Sulf-2 raises the possibility that it may be involved in promoting tumor progression [24,25]. Indeed, we have shown that Sulf-2 has pro-angiogenic activity, a relevant function for contributing to tumorigenesis [25].

Even before the discovery of the Sulfs, the glucosamine-6-sulfate modification of HS was the focus of considerable interest. Thus, it has been shown that suppressing the expression of heparan sulfate 6-O-sulfotransferases via gene silencing strategies has pronounced developmental consequences in both Drosophila and Zebrafish development [26,27]. Secondly, binding studies with heparin/HS fragments and chemically modified heparins point to an essential contribution of the glucosamine-6-sulfate modification to their interaction with various protein ligands, including FGF-1 [28], FGF-10 [29], PDGF [30], VEFG [31], hepatocyte growth factor [32], lipoprotein lipase [33], herpes simplex glycoprotein C [34], noggin [17], and L- and P-selectin [35]. Here, we have taken advantage of recombinant Sulf-2 generated in 293 cells and of native Sulf-2 obtained from conditioned medium of a breast carcinoma cell line to explore the range of activities for this enzyme. We have established an ELISA to examine the effects of Sulf-2 from these two sources on the interaction of several growth factors and chemokines with immobilized heparin/HS. We found that pre-treatment of heparin with Sulf-2 significantly reduced its reactivity with certain ligands but not others ("pre-binding effect") and the enzyme also dissociated heparin-ligand complexes ("post-binding effect") raising the possibility that it may be involved in mobilizing HS-bound ligands in the ECM and basement membranes.

## Results

### Effect of recombinant HSulf-2 on the binding of VEGF_165_, FGF-1 and FGF-2 to heparin

**Figure 1:**
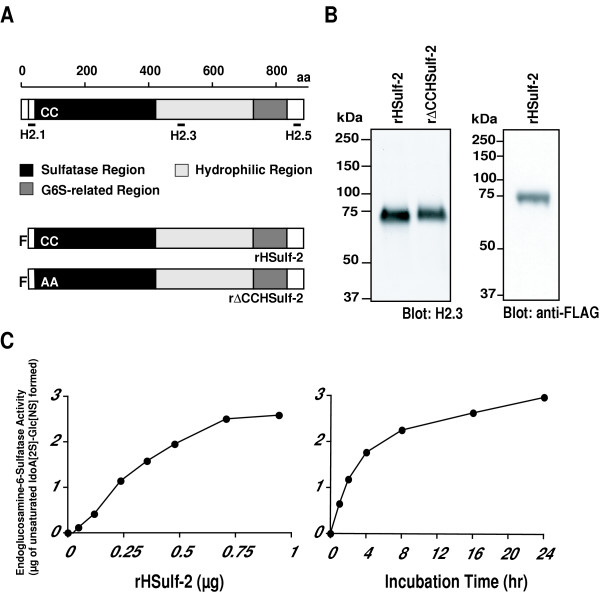
**Expression and characterization of a recombinant HSulf-2**. (A) Domains in HSulf-2 are schematically presented. Positions of peptides that are used to develop polyclonal antibodies are shown. A recombinant protein with a N-terminal FLAG-tag (F) (rHSulf-2) and an enzymatically inactivated protein (rΔCCHSulf-2) were designed and produced as described [25]. (B) Purified rHSulf-2 and rΔCCHSulf-2 produced in 293 cells were blotted with H2.3 anti-HSulf-2 antibody. A 72-kDa and a 74 kDa band were detected, respectively. rHSulf-2 was also blotted with a FLAG antibody. (C) Endoglucosamine-6-sulfatase activity of rHSulf-2 was measured as a function of enzyme concentration (**left panel**, 4 h incubation time) and reaction time (**right panel**, 240 ng of rHSulf-2) as described in **Methods**. rHSulf-2 liberated sulfate groups on C-6 position of glucosamine residues within trisulfated disaccharide units in heparin.

**Figure 2:**
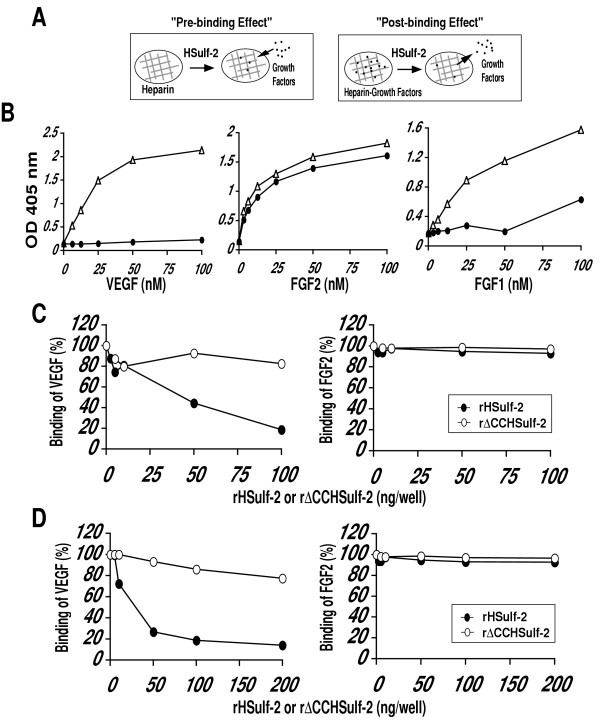
**Sulf-2 effects on pre- and post-binding assays for VEFG and FGF-2**. (A) Solid phase binding assays to measure "pre-binding effect" and "post-binding effect" are schematically shown. HSulf-2 was incubated with immobilized heparin before or after adding heparin-binding factors. Specific primary antibodies quantified the amount of factor bound to immobilized heparin. (B) Binding of ligand (VEGF_165_, FGF-2 or FGF-1) to immobilized heparin-BSA as a function of ligand concentration with or without Sulf-2 treatment of heparin-BSA. Δ denotes binding of protein to untreated heparin-BSA and ● denotes binding of VEGF, FGF-2 or FGF-1 to rHSulf-2 treated heparin-BSA in pre-binding assay. (C) "Pre-binding effect" of rHSulf-2 (●) or rΔCCHSulf-2 (○) on ligand binding (VEFG or FGF-2) to heparin-BSA as a function of enzyme concentration. (D) "Post-binding effect" of rHSulf-2 (●) or rΔCCHSulf-2 (○) on ligand binding (VEFG or FGF-2) to heparin-BSA as a function of enzyme concentration. All the results shown are representative of two independent experiments.

We wanted to know if Sulf-2, as a 6-O endosulfatase, could modulate the interaction of known heparin-binding growth factors to heparin. We prepared and purified amino-terminal FLAG-tagged HSulf-2 (rHSulf-2) from stably transfected kidney 293 cells (Figs. 1A and 1B). The major rHSulf-2 component detected by the H2.3 antibody migrated with an apparent molecular weight of 72 kDa. Immunoblotting of rHSulf-2 with a FLAG antibody revealed a protein band of the same molecular weight (Fig. 1B). We conclude that the 72 kDa component represents an N-terminal subunit with a C-terminal truncation. It should be noted that there are several potential furin cleavage sites that are C-terminal to the position of the peptide immunogen (aa 484–504) [15]. It is not known at present whether protein processing is necessary for enzymatic activity against heparan sulfate substrates. We also prepared an enzymatically-inactive mutant of HSulf-2 [15] in which two essential cysteines were mutated to alanines (rΔCCHSulf-2). As we had previously shown for CHO cell-produced material [15], rHSulf-2 exhibited endosulfatase activity against heparin in a concentration- and time- dependent manner (Figs. 1C and 1D). The assay employs HPLC to examine to the disaccharide composition of treated and untreated heparin (see **Methods**). After 24 h of enzyme treatment, 80% of the trisulfated disaccharides were converted to the disaccharide products. The mutant protein was inactive in this assay (data not shown). We next developed solid phase ELISAs to measure the binding of protein ligands to immobilized heparin-BSA. Antibodies directed to the ligands were used for detection. For "pre-binding" effects, we first treated heparin-BSA with rHSulf-2 and then tested for its ability to support ligand binding (Fig. 2A). For "post-binding" effects, we determined the ability of rHSulf-2 to release the ligand from a preformed complex with heparin-BSA (Fig. 2A).

We initially focused our attention on three growth factors: VEGF_165_, FGF-1 and FGF-2. Sulfate groups in heparin/HS, including glucosamine-6-sulfates, are required for the interaction of VEGF_165 _and FGF-1 with heparin/HS [28,31,36]. In contrast, FGF-2 binding to heparin/HS depends on N-sulfation and iduronate-2-sulfation but not on 6-O-sulfation [28,36,37]. We observed saturable binding of VEGF_165_, FGF-1 and FGF-2 to heparin-BSA (Figs. 2B and 4B). We estimated K_d_'s for these interactions and the binding of three chemokines (CXCL12, CCL21, and CXCL8) to heparin-BSA (Table 1). Negligible binding was seen when the wells were coated with BSA alone (data not shown). As a positive control to eliminate heparin-dependent binding, we pre-digested heparin-BSA with a mixture of heparinases (0.2 mU of heparinase I, 0.1 mU of heparinase II and 0.04 mU of heparinase III) for 4 h and found that this digestion reduced the binding of VEGF by >92%. Treatment of heparin with rHSulf-2 reduced the binding of varying concentrations of VEGF to heparin-BSA almost to background levels (Fig. 2B). rHSulf-2 treatment diminished the binding of FGF-1 by >60%. In contrast, rHSulf-2 had only a negligible effect on the binding of FGF-2 to heparin (Fig. 2B). To determine the efficacy of the enzyme, we fixed the concentration of ligand at approximately its K_d _and tested varying concentrations of rHSulf-2 or the inactive mutant, rΔCCHSulf-2. For FGF-2, only minimal effects of rHSulf-2 were seen up to 100 ng of the enzyme, whether tested in the pre-binding (Fig. 2C) or post-binding assay (Fig. 2D). In contrast, treatment of heparin-BSA with 50 ng or more of rHSulf-2 substantially reduced its binding to VEGF in a dose-dependent manner (Fig. 2C), whereas the mutant enzyme was largely inactive. In the post-binding assay, rHSulf-2 was also efficacious in releasing VEGF from preformed complexes with heparin-BSA (Fig. 2D, Table 2). The slight inhibitory effects of rΔCCHSulf-2 (Figs. 2C and 2D) in both the pre-binding and post-binding assays may be attributable to a competing heparin-binding activity of Sulf-2 protein due to its highly basic hydrophilic domain [15].

**Table 1:** Estimated dissociation constants for the interaction of ligands with immobilized heparin-BSA and heparan sulfate (HS)-BSA

Ligand	K_d _value (nM)	
	Heparin-BSA	HS-BSA

VEGF	12.5	>200
FGF-1	9.2	26
FGF-2	8.6	N.D.
CXCL12 (SDF-1)	75	N.D.
CCL21 (SLC)	5.5	N.D.
CXCL8 (IL8)	>180	N.D.

Each K_d _value was estimated from the concentration of ligand needed for half-maximal saturation. In the cases of IL-8 binding to heparin-BSA and VEGF binding to HS-BSA, saturation was not observed at the highest concentration tested.

N.D., not determined.

**Table 2:** Comparison of rHSulf-2 requirements for mobilization of heparin-bound ligands

Ligand	Concentration required for 50% release of heparin-bound ligand
	rHSulf-2 (μg/mL)

VEGF	0.34
FGF-1	0.36
FGF-2	>4.0
CXCL12 (SDF-1)	0.44
CCL21 (SLC)	3.1
CXCL8 (IL-8)	3.4
CXCL10 (IP-10)^a^	2.0

^a ^K_d _for heparin-BSA was 62 nM. The fixed concentration of ligand tested in the assay was 50 nM.

### Effects of HSulf-2 on chemokine binding to heparin-BSA

**Figure 3:**
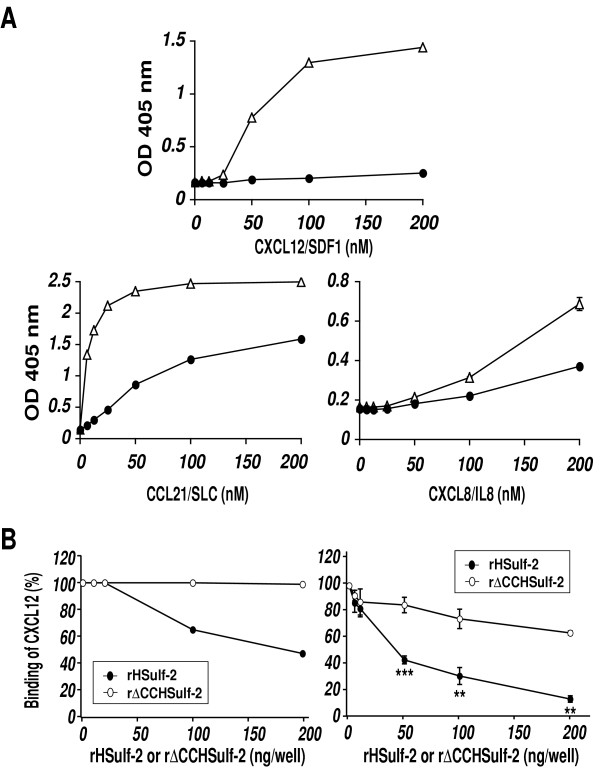
**Sulf-2 effects on pre- and post-binding assays for chemokines**. (A) Binding of CXCL12 (SDF-1), CCL21 (SLC) and CXCL8 (IL8) to immobilized heparin-BSA as a function of ligand concentration with or without Sulf-2 treatment of heparin-BSA. Δ denotes binding of chemokines to untreated heparin-BSA and ● denotes binding of chemokines to rHSulf-2 treated heparin-BSA in pre-binding assay. (B) "Pre-binding effect" (**left panel**) and "post-binding" effect (**right panel**) of rHSulf-2 (●) or rΔCCHSulf-2 (○) on ligand binding (CXCL12) to heparin-BSA as a function of enzyme concentration. Values represent the means ± S.D. of triplicate determinations in the binding assays for CXCL8 and CXCL12. Statistical analysis was carried out using Student's *t *test. **, p < 0.01; ***, p < 0.001. The other data are representative of two independent trials.

Chemokines are also heparin/HS-binding proteins [6]. However, limited information is available about the specific sulfation requirements for these interactions. We therefore took advantage of rHSulf-2 to determine the contributions of glucosamine-6-sulfation to these interactions. rHSulf-2 was very effective in reducing the interaction of SDF-1 (CXCL12) with heparin-BSA in both pre-binding and post-binding assays (Fig. 3A and 3B, Table 2). In contrast, rHSulf-2 had only partial inhibitory effects on the binding of SLC (CCL21) to heparin-BSA (Fig. 3A). IL-8 (CXCL8) bound less well to heparin-BSA, but rHSulf-2 treatment still attenuated the interaction (Fig. 3A).

### Analysis of the activity of native HSulf-2 in MCF-7 conditioned medium

**Figure 4:**
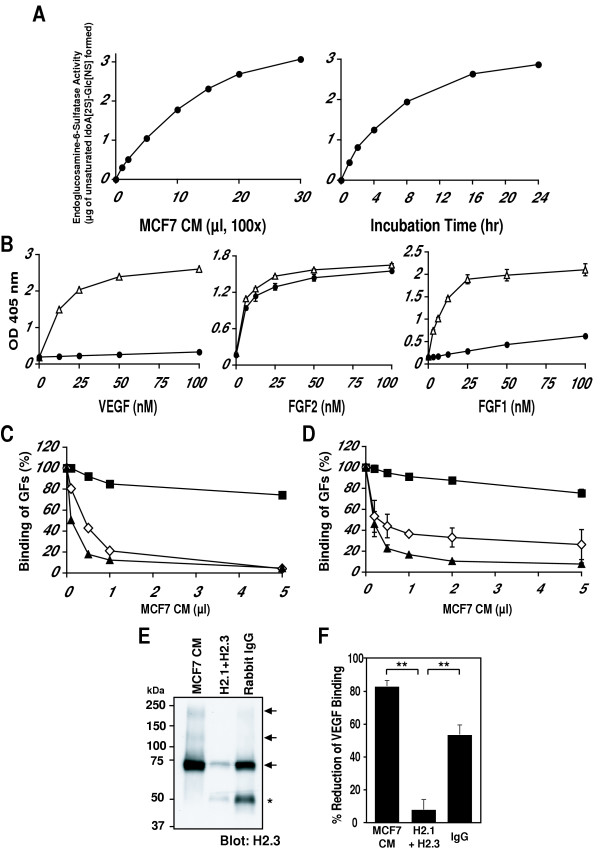
**Sulf-2 activity in MCF-7 CM**. (A) Endosulfatase activity of CM on heparin as a function of CM volume (**left panel**, 4 h incubation time) and reaction time (**right panel**, 10 μl of CM). (B) Binding of ligand (VEGF_165_, FGF-2 or FGF-1) to immobilized heparin-BSA as a function of ligand concentration with or without CM treatment of heparin-BSA. Δ denotes binding of ligand to untreated heparin-BSA and ● denotes binding of ligand to CM treated heparin-BSA in pre-binding assay. Means of triplicate determinations ± S.D. are shown. In those cases where the error bars are not apparent, the S.D. values are smaller than the symbols. (C) "Pre-binding effect" of CM on ligand binding (VEGF_165_, black triangles; FGF-2, black squares; FGF-1, open diamonds) as a function of CM volume. These results are representative of two independent experiments (D). "Post-binding effect" of CM on ligand binding (VEGF_165_, black triangles; FGF-2, black squares; FGF-1, open diamonds) as a function of CM volume. Means of triplicate determinations are shown. S.D. values for FGF-1 are indicated. The S.D. values for VEGF and FGF-2 are smaller than the symbols. (E) Immuno-depletion of HSulf-2 protein by specific antibodies. MCF-7 CM was precleared with Sulf-2 antibodies (H2.1 and H2.3) or control IgG conjugated to beads and then blotted for Sulf-2 protein (H2.3). Arrows denote specific bands detected in the untreated MCF-7 CM (~240, 135 and 72 kDa). The ~50 kDa bands indicated by the asterisk are derived from the IgG used for the precipitation (F). Immuno-depletion of HSulf-2 activity by specific antibodies. The precleared CMs in **E **were tested in a pre-binding assay for effects on VEGF binding to heparin-BSA. The partial depletion of activity by control IgG reflects the tendency of Sulf-2 to bind nonspecifically to Sepharose beads (not shown). Means and S.D. values are shown for triplicate determinations. Statistical analysis was carried out using Student's *t *test. **, p < 0.01.

*SULF2 *transcripts are upregulated in breast, central nervous system and colon carcinomas [24,25]. Moreover, transcripts are detected in some cultured human breast carcinoma cell lines and the mRNA-positive cells release Sulf-2 protein in their conditioned medium [25]. Sulf-2 expression is highest in the conditioned of MCF-7 cells and we have demonstrated that the secreted protein has associated arylsulfatase activity using 4-methylumbelliferyl sulfate as the substrate [25]. As shown in Fig. 4A, the CM from these cells exhibited concentration- and time-dependent endoglucosamine-6-sulfatase activity against heparin. In pilot experiments, the CM also showed activity against bovine intestinal heparan sulfate (HS) producing a substantial reduction in the amount of the trisulfated disaccharide with a corresponding increase in the amount of the disulfated disaccharide product (data not shown). Using CM as a source of the native enzyme, we analyzed the "pre-binding" and "post-binding" effects on the binding of VEGF or FGF-2 to heparin. Consistent with the rHSulf-2 findings, pretreatment of heparin-BSA with a fixed concentration of MCF-7 CM strongly reduced the subsequent binding of VEGF_165 _or FGF-1 but had no effect on the binding of FGF-2 (Fig. 4B). In a dose-dependent manner, CM prevented (Fig. 4C) and reversed (Fig. 4D) the interaction of VEGF_165 _or FGF-1 with heparin-BSA. Similar effects were observed with SDF-1 (data not shown). In contrast, the same range of CM concentrations had no appreciable effect on the binding of FGF-2 to heparin-BSA in either the pre-binding or post-binding assays (Figs. 4C and 4D). To confirm that the reductions in heparin binding were, in fact, due to Sulf-2, we employed anti-HSulf-2 antibodies [25] for immunodepletion of Sulf-2 from MCF CM. Pre-clearing was carried out by incubating CM with protein A-Sepharose complexed to a mixture of two specific antibodies and then centrifuging the mixture. As shown by Western blotting, the specific antibodies removed the majority of HSulf-2 protein from the CM, whereas control IgG removed only a relatively small amount of the protein (Fig. 4E). In parallel with the depletion of HSulf-2 protein, the "pre-binding" activity of the precleared CM, measured with respect to VEGF_165 _binding, was greatly diminished as compared to that of the control IgG-treated CM (Fig. 4F).

### Effects of HSulf-2 on heparan sulfate interactions

**Figure 5:**
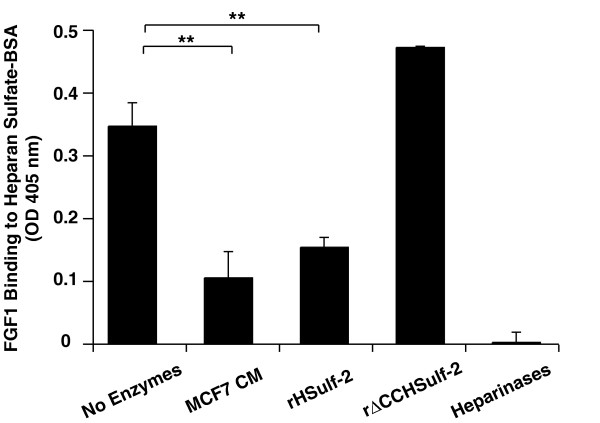
**The effect of Sulf-2 on FGF-1 binding to heparan sulfate-BSA**. FGF-1 binding to heparan sulfate-BSA was measured after the following treatments of the conjugate: no treatment; MCF-7 CM; rHSulf-2; rΔCCHSulf-2; or a mixture of heparinases. Values represent the means ± S.D. of three separate determinations. Statistical analysis was carried out using Student's *t *test. **, p < 0.01.

Heparin is a structural analogue of the S-domains of heparan sulfate. We wanted to determine whether the effects of Sulf-2 on heparin interactions would be repeated with bona fide heparan sulfate. For this purpose, we conjugated BSA with heparan sulfate chains (HS-BSA) that were isolated from porcine intestinal mucosa. FGF-1 bound to HS-BSA, although with a 3-fold lower affinity than to heparin-BSA (Fig. 5, Table 1). This binding was abolished by pre-digestion of the HS chains with heparinases. Pretreatment of the HS-BSA with MCF-7 CM or rHSulf-2 substantially diminished FGF-1 binding (55–70% reduction), whereas rΔCCHSulf-2 did not exert this effect (Fig. 5).

## Discussion

The Sulfs are 6-O endosulfatases that act on the trisulfated disaccharide unit (-IdoA(2-OSO_3_)-GlcNSO_3_(6-OSO_3_)-) which is the most common unit in heparin but is largely confined to the S-domains of HS [15-17]. As the heterogeneous pattern of sulfation within S domains is known to dictate the binding specificity of many proteins for heparin/HS [7], the Sulfs could potentially regulate those interactions with a dependence on the presence of trisulfated disaccharides within the binding motif. Here, we have employed Sulf-2 as a tool to explore the binding requirements of several proteins, some of which had been previously characterized and others whose binding requirements were largely unknown. Previous work has shown that QSulf-1 treatment of soluble recombinant form of an HSPG (Glypican-1) reduced its ability to bind a Wnt ligand [16]. Also, the interaction of Noggin with cell surface HSPGs was diminished by the overexpression of QSulf-1 in the cells [17]. In the present study we have taken advantage of two sources of soluble HSulf-2: recombinant enzyme purified from stably transfected 293 cells and a natural form of the enzyme in CM of a human breast carcinoma cell line (MCF-7). It should be noted that we have not been able to obtain expression of high level of Sulf-1 in CM of transfected 293 cells, nor have we yet identified a soluble form of this enzyme from a native source.

We have previously shown that native Sulf-2 in MCF-7 CM has arylsulfatase activity [25]. In the present study, we have used HPLC to demonstrate that MCF-7 CM possesses endosulfatase activity against intact heparin. This result has also been established by an alternative approach using mass spectroscopy [38]. Employing an ELISA based on binding of protein ligands to heparin-BSA conjugate, we found that rHSulf-2 strongly modulated the binding of VEGF_165_, FGF-1, and SDF-1 to porcine intestinal heparin. In the case of FGF-1, the inhibitory effect of Sulf-2 was also demonstrated for heparan sulfate chains as well, using a HS-BSA conjugate. All of the effects obtained with the recombinant enzyme were observed for MCF-7 CM, and we confirmed that the activity in the CM was due to Sulf-2. The porcine intestinal heparin used in this study contained 70% trisulfated disaccharides, 18% disulfated disaccharides, 7% monosulfated disaccharides and 5% unsulfated disaccharides (data not shown). Based on our previous work [15] and the present study, we estimate that about 80% of the total trisulfated disaccharides in heparin-BSA was converted into disulfated disaccharides by Sulf-2. Thus, only about 22% of the overall sulfate moieties were removed by the enzyme treatment, arguing against the possibility that the reduced binding of selective protein ligands to treated heparin was due to a reduction in overall charge. As heparan sulfates have limited S-domains [7], Sulf-2 would be predicted to have an even smaller effect on the global sulfation of HSPGs. A further indication of the selectivity of Sulf-2 is that there is no relationship between the estimated K_d _for binding of each ligand to heparin-BSA (Table 1) and the susceptibility of the interaction to the enzyme (Table 2).

The Sulf-2 effects on FGF-1 binding were anticipated based on a number of correlative studies [28,39], which have implicated the trisulfated disaccharide motif as a binding element in HS chains. Our VEGF_165 _results are compatible with a previous study in which chemical 6-O-desulfation of heparin strongly weakened its ability to interact with VEGF_165 _[31]. However, our findings provide the first direct evidence that 6-O-sulfation of heparin within the context of the trisulfated disaccharide motif is essential for the interaction. The majority of VEGF isoforms are able to bind to heparin/HS [40], and it is anticipated that our results will generalize to these other forms as well. With respect to SDF-1, it has long been known that this highly basic chemokine binds relatively strongly to heparin [41], but the fine specificity of the interaction has not previously been explored. SLC and IL-8 showed a partial sensitivity to the desulfation effects of Sulf-2 on heparin. This partial susceptibility implies that the 6-O-sulfation of the trisulfated disaccharide can contribute but is not absolutely required for a measurable binding interaction with heparin. One important avenue for future investigation is based on our observation that Sulf-2 strongly modulated the interactions of two chemokines (SDF-1 and SLC) with heparin. SDF-1 has been implicated in a diverse range of processes such as lymphocyte chemotaxis, stem cell homing and retention, tissue repair, angiogenesis, and organ-specific metastasis [42], whereas SLC is important in lymphoid organ homeostasis and inflammation [43]. Thus, Sulf-2 though effects on the ECM-association of chemokines could have important roles in a number of normal and pathophysiologic processes. HSulf-1 with an apparently indistinguishable enzymatic activity [15] may exert similar effects on HSPG-bound growth factors and chemokines.

It is well established that one function of heparan sulfate proteoglycans in the ECM and basement membranes is to sequester protein ligands away from signal transduction receptors [44,45]. A known mechanism to regulate the mobilization of such factors is through the action of heparanase, an endo-ß-D-glucuronidase that degrades heparan sulfate chains into relatively large fragments [46]. This enzyme is present in a number of normal cell types (e.g., leukocytes, platelets, cytotrophoblasts) and is upregulated in several cancers. Expression of heparanase in a number of settings elicits angiogenesis, apparently through the release of HSPG-bound angiogenic factors [44,47]. Our finding that Sulf-2 can reverse the association between heparin and angiogenic factors (e.g., VEGF) suggests the possibility of functional parallels between these two enzymes. Sulf-2 may mobilize VEGF or other angiogenic factors sequestered in the ECM and increase their bioavailability to endothelial cells that express the appropriate signaling receptors. The ability of rHSulf-2 to promote angiogenesis in the chick chorioallantoic membrane (CAM) assay is consistent with such a scenario [25]. Thus, the observed upregulation of Sulf-2 in mammary carcinomas and its secretion from these cells could directly contribute to tumor angiogenesis and thus tumor growth. Sulf-2 derived from a cancer cell could also conceivably mobilize HSPG-bound growth factors that then act back on the cancer cell and thus trigger its own proliferation. The ability of QSulf-1 to promote Wnt signaling within the cells that express the enzyme provides a plausible paradigm for this type of autocrine pathway [14,16]. It should be borne in mind that in the context of some other signaling pathways (e.g., FGF-2 and HGF), Sulf-2 is likely to exert a negative effect.

## Conclusion

As demonstrated in the present study, Sulf-2 can modulate the interaction of a number of important bioactive proteins with heparin/HS. A major finding of our study is that Sulf-2 completely disrupts the interaction of certain factors with heparin. As expected, the factors for which Sulf-2 strongly blocks binding to heparin in the pre-binding format are the most susceptible to reversal effects in the post-binding assays (VEGF, FGF-1, and SDF-1). These observations indicate that, irrespective of the engagement of a protein ligand, Sulf-2 has access to the 6-O-sulfate group of the trisulfated disaccharide units of intact heparin. Recombinant Sulf-2 provides a valuable tool for investigating the specific sulfation requirements for heparin/HS interactions of interest. Furthermore, future application of the assays described herein will certainly expand the range of interactions that can be modulated by Sulf-2 and should enhance our understanding of the potential functional roles served by this enzyme in situ.

## Methods

### Materials

The following materials were obtained commercially from the source indicated. Heparin conjugated with bovine serum albumin (Heparin-BSA), BSA, heparinases (I, II and III) were from Sigma (St Louis, Mo., USA); heparan sulphate (porcine intestinal mucosal) from Organon (Oss, The Netherlands); DEAE-Sephacel from Sigma (Poole, UK); Phenyl-Sepharose CL-4B from Amersham Biosciences (Little Chalfont, UK); Protein A Sepharose beads from RepliGen (Needham, MA); recombinant human VEGF_165_, human CXCL12 (SDF-1), anti-human VEGF antibody (Ab), anti-human FGF-1 Ab, anti-human FGF-2 Ab, anti-human CXCL12 Ab, anti-CXCL8 Ab and anti-human CCL21 Ab from R&D Systems. All are polyclonal antibodies produced in goats. Recombinant human FGF-1 (acidic FGF), human FGF-2 (basic FGF), human CCL21 (SLC) and CXCL8 (IL-8) were from Leinco Inc. A biotinylated swine anti-goat IgG (H+L) antibody and a streptavidin conjugated with alkaline phosphatase were from Caltag. FLAG-tagged versions of HSulf-2 and the inactive mutant designated ΔCCHSulf-2 were produced in 293 cells and purified as previously described [25]. Polyclonal rabbit anti-bodies against HSulf-2 (H2.1 and H2.3) were generated and prepared as previously described [25].

### Heparan sulfate-BSA conjugate

Porcine intestinal mucosal heparan sulfate was coupled, via its reducing end, to BSA based on the procedure of Najjam et al. [48]. Briefly, 2 mg of sodium cyanoborohydride was added to a mixture of 50 mg of HS and 2 mg of BSA in 2 ml of 0.2 M potassium phosphate buffer pH 8. The mixture was then incubated for 2 days at 37°C. After dilution to 10 ml with 0.15 M NaCl, 20 mM phosphate, pH7.4, it was applied to a 5 ml column of DEAE-Sephacel equilibrated in the same solution. After washing with 0.15 M NaCl solution, followed by buffered 0.4 M NaCl, to remove reagents and any non-conjugated BSA, the HS-containing material was then step-eluted with 0.5 ml aliquots of buffered 1.5 M NaCl. HS-containing fractions were identified by spotting aliquots onto Whatman filter paper and staining with Azure A. The pooled fractions were adjusted to 3 M in (NH_4_)_2_SO_4, _by addition of solid (NH_4_)_2_SO_4_, and then applied to a 2 ml column of Phenyl-Sepharose CL-4B equilibrated in 3 M (NH_4_)_2_SO_4_. After washing with 3 M (NH_4_)_2_SO_4 _to elute free HS, the bound HS-BSA conjugate was step-eluted with 0.5 ml aliquots of 0.15 M NaCl, 20 mM phosphate pH7.4. Fractions containing conjugated BSA were identified by their absorbance at 280 nm. The final product ran as a diffuse band, stainable with both Coomassie Blue and Azure A, close to the top of a 10% polyacrylamide SDS-PAGE gel and with a higher apparent MW than free BSA. The BSA content of the conjugate was assessed by absorbance at 280 nm. The HS content was determined by digestion with a mixture of heparinases I, II and III, and measurement of the resulting increase in absorbance at 232 nm. It was estimated that the average level of conjugation was 1–2 HS chains/BSA molecule.

### Preparation of recombinant Sulf-2 and CM from MCF-7 cells

FLAG-tagged versions of active Sulf-2 (rHSulf-2) and inactive enzyme (rΔCCHSulf-2) were produced in stably transfected kidney 293 cells as described previously [25]. The purities of rHSulf-2 and rΔCCHSulf-2 were estimated to be 90% and 80%, respectively based on Coomassie blue staining of SDS-PAGE gels. The amount of Sulf-2 protein was quantified by the BCA assay (Pierce). The amounts of rHSulf-2 and rΔCCHSulf-2 were equalized by an ELISA employing an anti-FLAG M2 antibody (Sigma) as the capture reagent and a mix of H2.1 and H2.3 antibodies for detection. The human breast carcinoma cell line, MCF-7, was grown in RPMI-1640 containing 10% fetal calf serum in a 150 mm-flask. Cells (80% confluent) were rinsed with PBS and OptiMEM (Invitrogen) and then incubated in 25 ml of OptiMEM at 37°C in 5% CO_2 _for 72 h. The CM was collected and then 100-fold concentrated on a Centricon-30. Subsequently, the concentrated CM was dialyzed into 50 mM HEPES, pH 8.0.

### ELISAs for "pre-binding" and "post-binding" effects of HSulf-2

The experimental design for these ELISA is schematically shown in Fig 2A. To immobilize heparin, 100 ng/ml of heparin-BSA in phosphate-buffered saline (PBS) was added to the wells (100 μl/well) of a 96-well plate (Immulon 2HB, Dynex Labs). The plate was placed at 4°C overnight. The wells were washed 3 times with PBS containing 0.1% Tween-20 (PBS-T) and then blocked with 3% BSA (Sigma) in PBS-T at 25°C for 2 h. To determine the effects of Sulf-2 on the ligand-binding activities of heparin ("pre-binding" effects), the wells were washed as above and then incubated with 100 μl of a reaction mixture containing 5 μmol of HEPES, pH 7.6, 1 μmol of MgCl_2 _and enzyme (rHSulf-2, rΔCCHSulf-2 or MCF-7 CM) at 37°C for 4 h. In the cases where we carried out dose response studies with protein ligands, incubation with the enzyme was overnight. The wells were washed 3 times with PBS-T and then incubated with 25 μl of heparin-binding factors (25 nM VEGF, 12.5 nM FGF-2, 12.5 nM FGF-1, and 100 nM CXCL12) in PBS at 25°C for 30 min. The wells were washed 3 times and incubated with 1 μg/ml of each primary antibody in 0.1% BSA/PBS (100 μl/well) at 25°C for 1 h. The wells were washed as above and incubated with 1.2 μg/ml of a biotinylated secondary antibody in 0.1% BSA/PBS (100 μl/well) at 25°C for 30 min. Then, the wells were washed and incubated with 2 μg/ml of an alkali phosphatase-conjugated streptavidin in 0.1% BSA/PBS (100 μl/well) at 25°C for 30 min. Finally, the wells were incubated with PNPP (Pierce) in cacodylate buffer, pH 9.2 (100 μl/well) at 25°C for 5 minutes, and then OD 405 nm was read on a microplate reader. For the analysis of "post-binding" effects (i.e. dissociation of heparin-ligand complexes), 25 μl of each heparin-binding factor was added to plates coated with heparin-BSA after blocking with 3% BSA. The plates were incubated at 25°C for 30 min to form the heparin-factor complex. The wells were washed as above and then incubated with 100 μl of the enzyme reaction mixture as described above. After washing the wells, the amount of bound factor was determined with the antibody detection procedures described above. For the "pre-binding" study of FGF-1 binding to heparan sulfate-BSA, 1 mg/ml of heparan sulfate-BSA was coated on a 96-well plate (100 μl/well). Other procedures are the same as described above for the heparin-BSA assay. Representative data are shown from one of 2–3 independent experiments in all cases.

### Western blotting

A purified FLAG-tagged HSulf-2 (10 ng) or concentrated MCF-7 CM (10 μl) was separated by electrophoresis on a reducing SDS-7.5% polyacrylamide gel (Bio-Rad), and blotted onto ProBlott™ membrane (Applied Biosystems, Foster City, CA). The membrane was blocked with 5% skim milk/PBS-T for 1 h and then incubated overnight with an anti-HSulf-2 peptide antibody (see below) at a concentration of 1 μg/ml in 5% skim milk/PBS-T at 4°C. The membrane was washed and incubated with horseradish peroxidase-conjugated goat anti-rabbit IgG (Jackson ImmunoResearch Laboratories Inc, West Grove, PA) (0.2 μg/ml) for 1 h. Bound antibodies were visualized with SuperSignal West Pico Chemiluminescent reagent (Pierce).

### Immunoprecipitation of HSulf-2 in MCF-7 CM

A mixture of anti-HSulf-2 peptide antibodies, H2.1 (5 μg) and H2.3 (5 μg) [25], or a rabbit IgG (10 μg), 10 μl of a 50% (v/v) suspension of protein A-Sepharose (IPA-400HC, RepliGen) and 100 μl of 100-fold concentrated CM of MCF-7 cells were mixed in a total volume of 1 ml with 150 mM NaCl and 50 mM Tris-HCl, pH 7.4. The mixture was rocked overnight at 4°C. The immunocomplexes bound to the protein A beads were removed by centrifugation. The "pre-binding" and "post-binding" ELISA activities remaining in the supernatant were assayed as described above.

### Endoglucosamine-6-sulfatase assay

Endoglucosamine-6-sulfatase activity of rHSulf-2 and MCF-7 CM was determined by previously established procedures [15]. The standard reaction mixture contained 5 μmol of HEPES, pH7.8, 1 μmol of MgCl_2_, 5 μg of porcine intestinal heparin (Sigma), and a purified FLAG-tagged HSulf-2 or a 100-fold concentrated MCF-7 CM in a total volume of 100 μl. The mixture was incubated at 37°C. The reaction was stopped by heating at 100°C for 2 min. The heparin was digested into disaccharides by a mix of heparinase I, heparinase II and heparinase III [15]. The disaccharides were then analyzed by HPLC on a Partisil-10 SAX column (Whatman, Fairfield, NJ) [15]. The enzymatic activity was defined by calculating the increase in the moles of unsaturated disulfated disaccharide, IdoA(2S)-Glc(NS), in the digested heparin.

## List of abbreviations used

CHO, Chinese hamster ovary, CM, conditioned medium; ECM, extracellular matrix; FGF, fibroblast growth factor; GAG, glycosaminoglycan; HS, heparan sulfate; HSPG, heparan sulfate proteoglycan; HSulf-2, human Sulf-2; rHSulf-2, recombinant flag-tagged human Sulf-2; rΔCCHSulf-2, recombinant inactive mutant of human Sulf-2; SAGE, serial analysis of gene expression; SDF, stromal cell-derived factor; VEGF, vascular endothelial growth factor.

## Authors' contributions

KU participated in the study design and coordination, performed and interpreted the studies, and drafted the manuscript. MM-T and AB participated in the studies of recombinant HSulfs and preparation of anti-HSulf antidodies. JL participated in ELISA studies. ML and JG participated in the studies with heparan sulfate-BSA. ZW participated in the study design. SDR participated in the design and coordination of the studies and edited the manuscript. All authors have read and approved the final manuscript.
